# Supplementary material for: DAF‐2/insulin IGF‐1 receptor regulates motility during aging by integrating opposite signaling from muscle and neuronal tissues
Source: Aging Cell. 2022 Jul 8;21(8):e13660. doi: 10.1111/acel.13660 (PMC9381905; doi:10.1111/acel.13660)
Supplement: Supplementary file 1 — Figures S1‐S4 [file ACEL-21-e13660-s001.pdf]

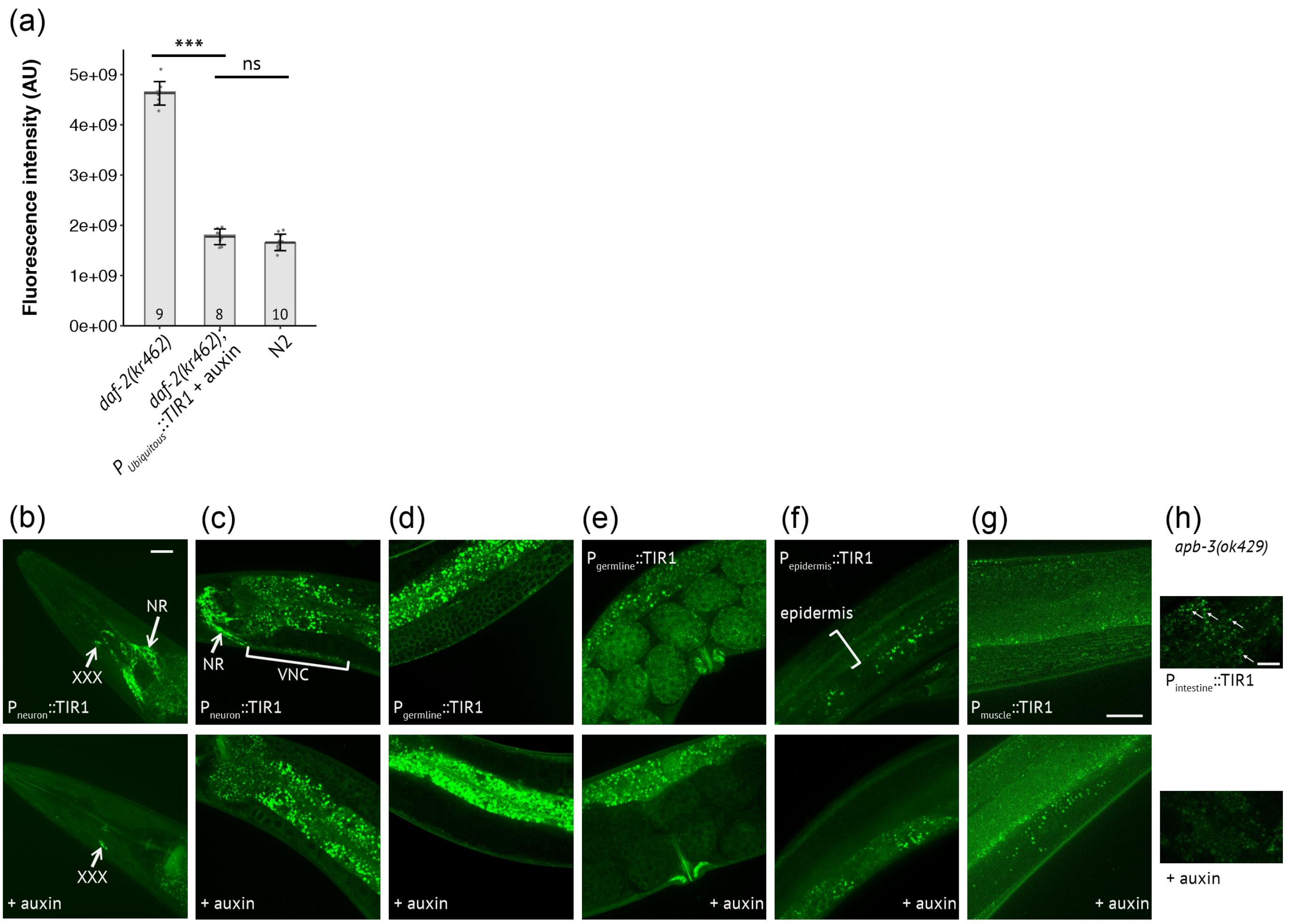

Supplementary Figure S1: DAF-2::AID::mNG is efficiently degraded in different tissues in presence of auxin.
(**a)** Quantification of fluorescence associated with DAF-2::AID::mNG expression in the absence or presence of 1mM auxin as indicated. Worms were transferred to auxin-containing plates at the L4 stage. Twenty-four hours later, the heads of the animals were imaged and quantified (see Experimental procedures). N2 were used as control for nonspecific autofluorescence. The numbers of animals scored is indicated in bars. Bars indicate median values, means are represented by black horizontal lines and brackets show standard deviations, ns: non-significant, ***: $p < 0.001$, KruskalWallis and Dunn's post hoc test with FDR method for adjusting p-value. **(b-h)** Images of DAF-2::AID::mNG expression in 1-day-old animals. Animals express transgenic TIR1 under the control of tissue specific promoters, as indicated, in the absence (upper panel) or in the presence (lower panel) of 1 mM auxin for 24 hours. **(a-b)** With a neuronal promoter, DAF-2::AID::mNG was no longer detected in the nerve ring (NR) **(b)** and in the ventral nerve cord (VNC) **(c)** but was still detected in the XXX cell **(b)**. **(d,e)** With a germline promoter, DAF-2::AID::mNG signal was reduced in the proliferating germ cells **(d)** and in the eggs **(e)**. **(f)** With a epidermal promoter, DAF-2::AID::mNG disappeared from the epidermis syncytium. **(g)** With a muscular promoter, DAF-2::AID::mNG was no longer detected in body wall muscles. **(h)** With an intestinal promoter, DAF-2::AID::mNG signal was downregulated in the intestine. *apb-3(ok429)* mutants with reduced intestinal autofluorescence were used. Scale bars: 20 µm **(b-g)**, 10 µm **(h)**.

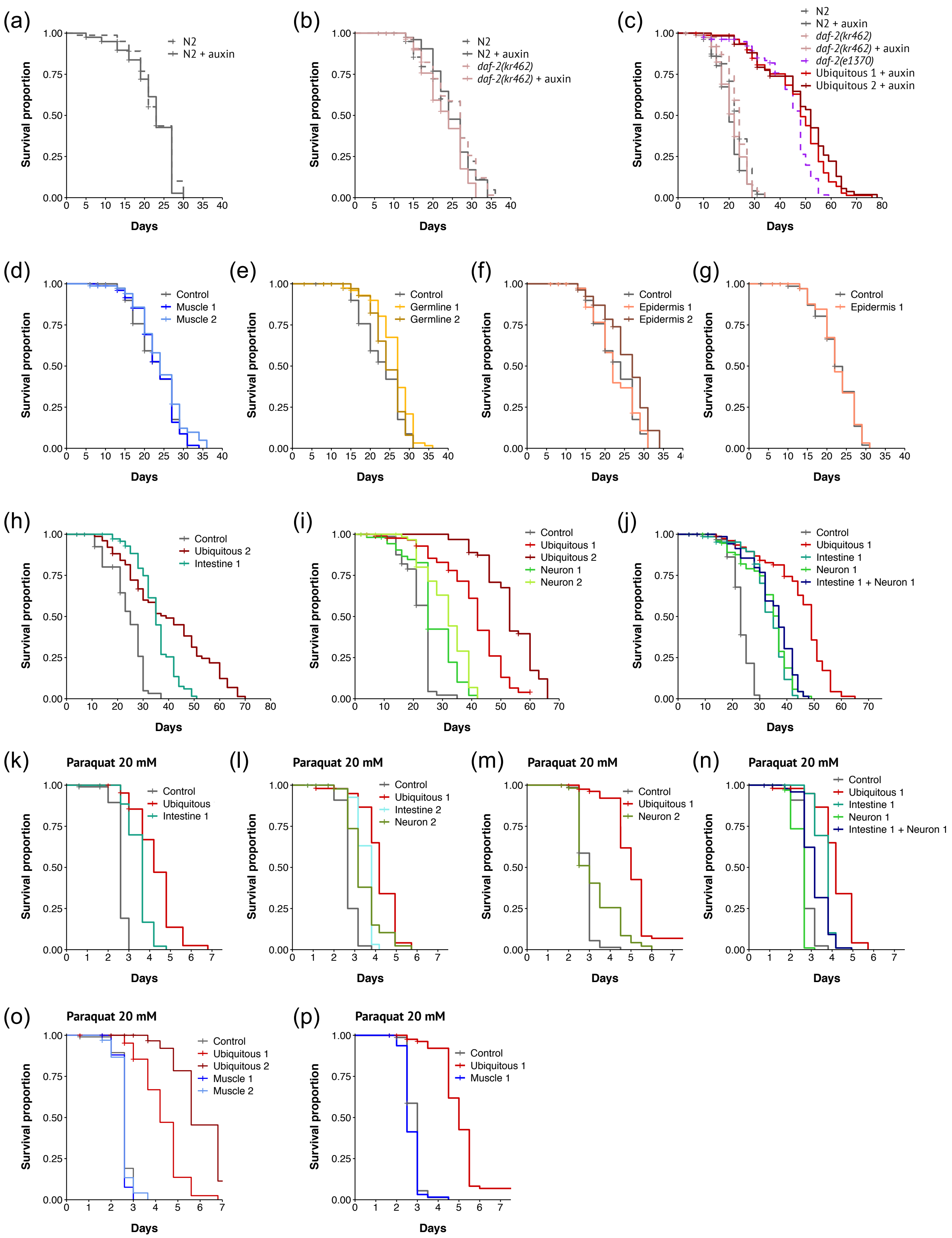

Supplementary Figure 2: Survival curve of biological replicates corresponding to Table S2.
Survival curves of animals with DAF-2 depletion in none, all cells or in the indicated tissue in absence **(a-j)** or in presence of 20 mM paraquat (**k-p**). All conditions corresponding to (**d-p**) were in presence of 1mM auxin. For detailed genotypes and statistics, see Table S2.

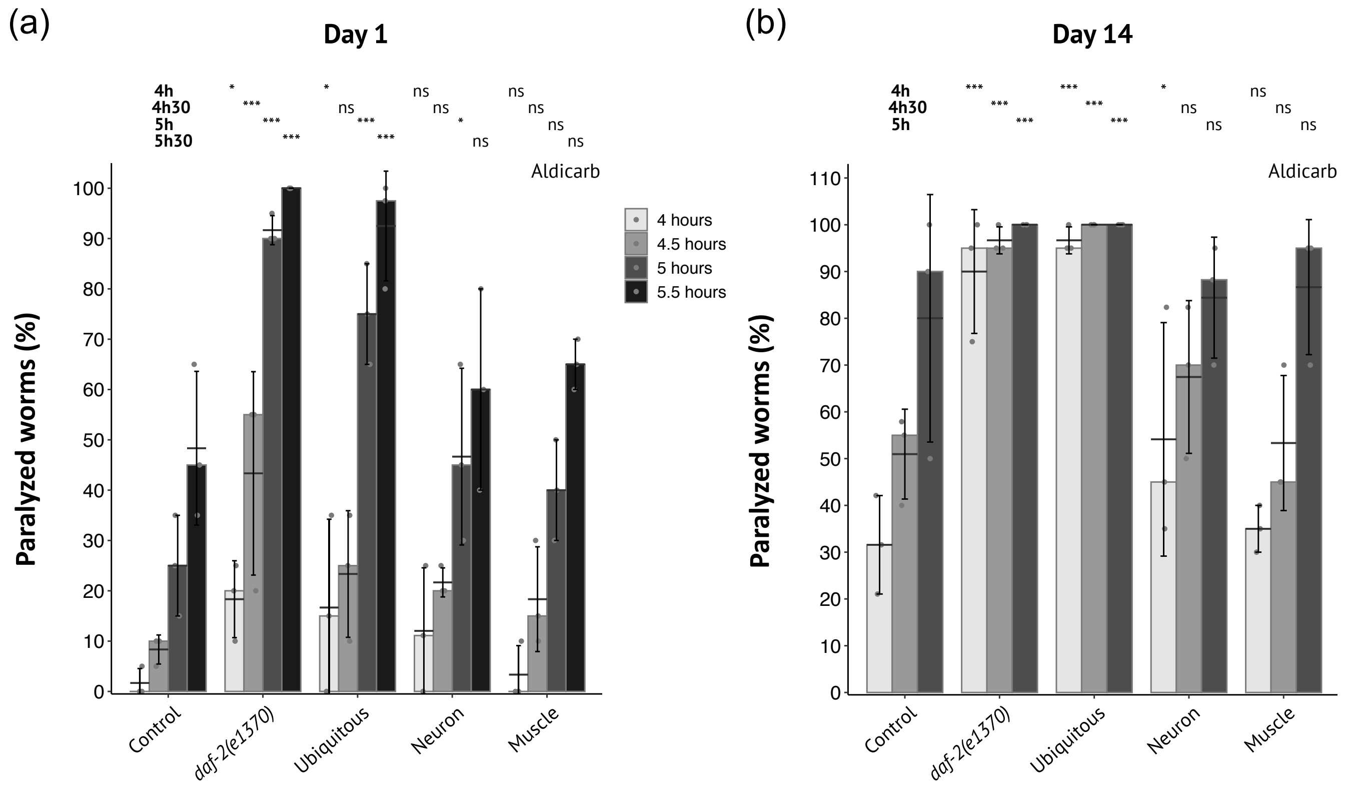

Supplementary Figure 3: Ubiquitous DAF-2 depletion induced aldicarb hypersensitivity.
Synchronized 1-day-old **(a)** or 14-day-old **(b)** adults with ubiquitous (ubiquitous 1), neuronal (neuron 2) or muscle (muscle 1) DAF-2 depletion from the L4 stage, or *daf-2(e1370)* mutants, as indicated, were placed in a drop of M9 containing 250 μM aldicarb. Control corresponds to *daf-2(kr462)* worms cultivated in presence of auxin from the L4 stage. The cholinesterase inhibitor aldicarb causes an accumulation of acetylcholine in the synaptic cleft of neuromuscular junctions, resulting in sustained muscle activation and eventually paralysis. Worms were assayed for paralysis over time (see Experimental procedure). Data from 3 independent experiments have been pooled (n= 60 individuals per condition). Comparisons used Fisher exact test, followed by pairwise tests with FDR adjusting method as post-hoc tests: ns: not significant, *: $p_{adjusted} < 0.05$, ***: $p_{adjusted} < 0.001$. Statistics are presented in several lines corresponding to the comparison with the control strain at each time point.

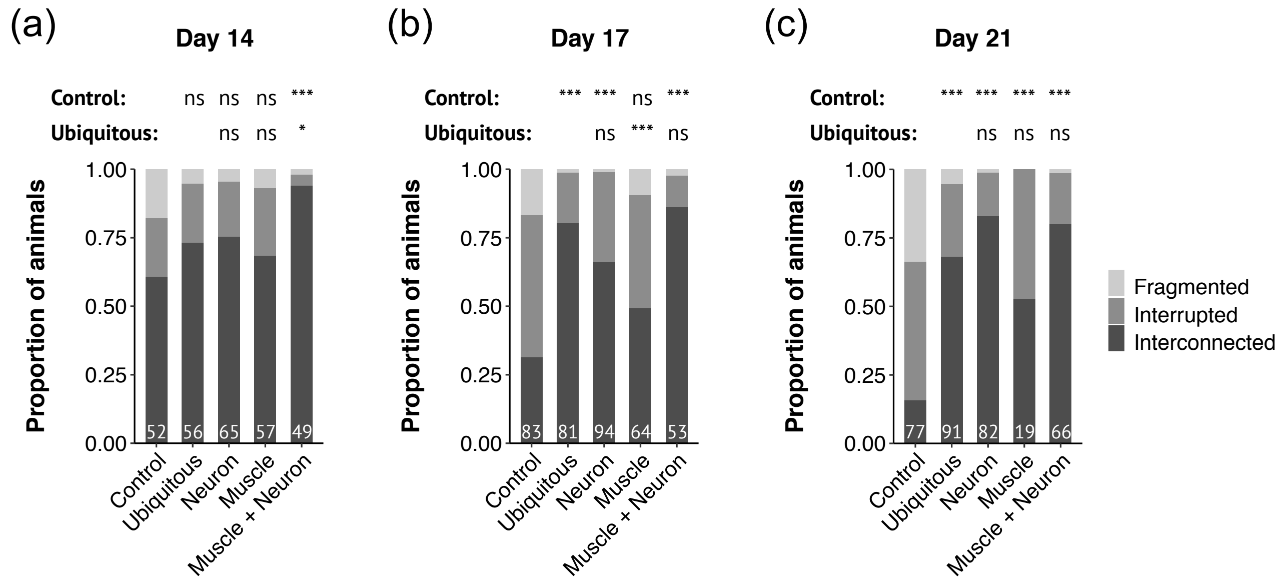

Supplementary Figure 4: Muscle or neuronal depletion of DAF-2 is sufficient to prevent muscle mitochondria fragmentation during ageing

(a-c) Quantification of muscular mitochondrial morphology at day 14 **(a)**, day 17 **(b)**, or day 21 **(c)** of adulthood in worms with DAF-2 depletion in all tissues, or in muscle, neurons or muscle and neurons as indicated. The number of animals scored is indicated in each bar. Data correspond to a pool of 3 to 5 independent trials depending on the strain. Strains used were: *daf-2(kr462)* in presence of auxin as Control, Ubiquitous 1, Neuron 1 and 2, Muscle 1, Muscle 1 + Neuron 1 (see Table S1 for strain description). Comparisons used Fisher exact test, followed by pairwise tests with FDR adjusting method as post-hoc tests: ns: not significant, *: $p_{adjusted} < 0.05$, ***: $p_{adjusted} < 0.001$. Statistics are presented as two lines that include comparison with the control and Ubiquitous 1 strain.
