# Supplementary material for: DAF‐2/insulin IGF‐1 receptor regulates motility during aging by integrating opposite signaling from muscle and neuronal tissues
Source: Aging Cell. 2022 Jul 8;21(8):e13660. doi: 10.1111/acel.13660 (PMC9381905; doi:10.1111/acel.13660)
Supplement: Supplementary file 2 — Tables S1‐S5 [file ACEL-21-e13660-s002.pdf]

Supplemental experimental procedures

Dauer, lifespan and oxidative stress assays

For dauer assays, L4s were transferred to ethanol or 1 mM auxin plates and allowed to mature into egg-laying adults, then removed after twenty-four hours. Progeny were assessed after forty-eight hours at 25°C for the presence of dauer larvae.
For lifespan assays, cohorts were handled as described in "aging cohorts". Lifespans were conducted at 20°C and were assessed every 2–3 days as described previously (Masse et al., 2008). Briefly, animals were scored as dead when they stopped moving and responding to prodding, they were censored when they crawled off the plate, had a "protruding vulva" or an "exploded vulva". For oxidative stress survival assays, animals were transferred to 1 mM auxin plates at the L4 stage. After seven hours, young adult animals were transferred to 1 mM auxin plates supplemented with 20 mM paraquat (Methyl viologen dichloride hydrate, Sigma-Aldrich). Animals were scored as in lifespan assays but twice a day. All lifespan tests were blinded to avoid bias in the evaluation.

Alleles generation by CRISPR/Cas9 genome engineering

To generate the *kr462* allele, a flexible linker, the AID sequence (Zhang et al., 2015), another flexible linker and mNeonGreen were inserted tandemly into the *daf-2* locus, just before the stop codon. This sequence was PCR amplified with or without homology arms from pCR12 and the PCR products were used to generate the repair template (Dokshin et al., 2018). To generate the *kr535* allele, a flexible linker, the wrmScarlet, another flexible linker and 3 MYC tags were inserted tandemly into the *daf-16* locus, just before the stop codon. This sequence was PCR amplified with or without homology arms and the PCR products were used to generate the repair template (Dokshin et al., 2018). CrRNA were designed on *Benchling.com* and synthesized by IDT (Integrated DNA Technologies). For *kr462* TGAAAATGAGCATCTAATCG and ttttgggggttTCAGACAAG crRNA were used in tandem, for *kr535* CATGAGCTGAGTCAAGCTGG and tctctttcgaacaacaccag were used in tandem. The injection mix contained annealed double-stranded DNA donor cocktail as repair template at 200 ng/µL, Cas9 nuclease at 0.25 µg/µL (Integrated DNA Technologies), tracrRNA-crRNA duplex at 9 µM, pRF4 [*Peft-3::rol-6*] co-injection marker at 2.5 ng/µL, and RNase/DNase-free water qsp 20 µL. The candidate F1 animals were isolated by tracking the initial fluorescence knock-in in plates with F1 roller progeny. The F2 progenies were then isolated and homozygosed. The insertion was then confirmed by PCR and sequencing. Candidates were then outcrossed once with N2 before further crosses to generate the desired strains (See Table S1).

RNAi

Bacterial feeding RNAi experiments were carried out essentially as described previously (Masse et al., 2008). Briefly, single colonies of bacteria containing plasmids of interest were first grown overnight in LB with 100 mg/mL ampicillin and 12.5 mg/mL tetracycline and then for about 5 h in LB with 100 mg/mL ampicillin until OD600 reached 0.6 to 0.8. RNAi bacterial cultures were concentrated 6 times in order to increase their efficacy. Bacteria were seeded onto NGM plates containing 2 mM IPTG, 25 mg/mL carbenicillin and 1mM auxin. Worms were maintained on OP50 plates and transferred on RNAi plates at the L4 stage. The control (HT115) and D1081.2 (*unc-120*) clones were purchased from GeneService. Each clone has been sequenced to confirm its identity. In all RNAi experiments *rrf-3(pk1426*) mutant worms were used in order to increase RNAi sensitivity (Simmer et al., 2003).

Supplementary references

Ashley, G. E., Duong, T., Levenson, M. T., Martinez, M. A. Q., Johnson, L. C., Hibshman, J. D., Saeger, H. N., Palmisano, N. J., Doonan, R., Martinez-Mendez, R., Davidson, B. R., Zhang, W., Ragle, J. M., Medwig-Kinney, T. N., Sirota, S. S., Goldstein, B., Matus, D. Q., Dickinson, D. J., Reiner, D. J. & Ward, J. D. (2021). An expanded auxin-inducible degron toolkit for *Caenorhabditis elegans* D. Greenstein, ed. *Genetics* 217, iyab006. https://10.1093/genetics/iyab006

Bhattacharya, A., Aghayeva, U., Berghoff, E. G. & Hobert, O. (2019). Plasticity of the Electrical Connectome of C. elegans. *Cell* 176, 1174-1189.e16. https://10.1016/j.cell.2018.12.024

Dokshin, G. A., Ghanta, K. S., Piscopo, K. M. & Mello, C. C. (2018). Robust Genome Editing with Short Single-Stranded and Long, Partially Single-Stranded DNA Donors in *Caenorhabditis elegans*. *Genetics* 210, 781–787. https://10.1534/genetics.118.301532

Hills-Muckey, K., Martinez, M. A. Q., Stec, N., Hebbar, S., Saldanha, J., Medwig-Kinney, T. N., Moore, F. E. Q., Ivanova, M., Morao, A., Ward, J. D., Moss, E. G., Ercan, S., Zinovyeva, A. Y., Matus, D. Q. & Hammell, C. M. (2022). An engineered, orthogonal auxin analog/ *At* TIR1(F79G) pairing improves both specificity and efficacy of the auxin degradation system in *Caenorhabditis elegans* H. Buelow, ed. *Genetics* 220, iyab174. https://10.1093/genetics/iyab174

Masse, I., Molin, L., Mouchiroud, L., Vanhems, P., Palladino, F., Billaud, M. & Solari, F. (2008). A Novel Role for the SMG-1 Kinase in Lifespan and Oxidative Stress Resistance in Caenorhabditis elegans A. C. Hart, ed. *PLoS ONE* 3, e3354. https://10.1371/journal.pone.0003354

Mergoud dit Lamarche, A., Molin, L., Pierson, L., Mariol, M. C., Bessereau, J. L., Gieseler, K. & Solari, F. (2018). UNC-120/SRF independently controls muscle aging and lifespan in *Caenorhabditis elegans*. *Aging Cell* 17, e12713. https://10.1111/acel.12713

Sijen, T., Fleenor, J., Simmer, F., Thijssen, K. L., Parrish, S., Timmons, L., Plasterk, R. H. A. & Fire, A. (2001). On the Role of RNA Amplification in dsRNA-Triggered Gene Silencing. *Cell* 107, 465–476. https://10.1016/S0092-8674(01)00576-1

Simmer, F., Moorman, C., van der Linden, A. M., Kuijk, E., van den Berghe, P. V. E., Kamath, R. S., Fraser, A. G., Ahringer, J. & Plasterk, R. H. A. (2003). Genome-Wide RNAi of C. elegans Using the Hypersensitive rrf-3 Strain Reveals Novel Gene Functions James Carrington, ed. *PLoS Biol* 1, e12. https://10.1371/journal.pbio.0000012

Zhang, L., Ward, J. D., Cheng, Z. & Dernburg, A. F. (2015). The auxin-inducible degradation (AID) system enables versatile conditional protein depletion in *C. elegans*. *Development*, dev.129635. https://10.1242/dev.129635

Zhou, X., Vachon, C., Cizeron, M., Romatif, O., Bülow, H. E., Jospin, M. & Bessereau, J. L. (2021). The HSPG syndecan is a core organizer of cholinergic synapses. *Journal of Cell Biology* 220, e202011144. https://10.1083/jcb.202011144

Table S1: Strains

Strains	Genotypes	Promotor driving TIR1 expression	Tissues with DAF-2 degradation	Source of TIR1 alleles	Used in figure(s)
**EN7563**	*kr462[daf-2::linker::AID::linker::mNeonGreen] III; krSi50[Peft-3::TIR1::tagBFP::unc-54 3' UTR] IV*	*eft-3*	Ubiquitous 1	Zhou et al., 2021	1 (c, d, e); 2; 3; 4 (a, c); S1 (a); S2; S3
**EN7863**	*kr462 III; krSi45[Peft-3::TIR1::tagBFP::unc-54 3' UTR] V*	*eft-3*	Ubiquitous 2	This work	1 (c); 3; 4 (a, c); S2
**EN7565**	*kr462 III; krSi55[Pmyo-3::TIR1::tagBFP::unc-54 3' UTR] V*	*myo-3*	Muscle 1	Zhou et al., 2021	3; 4 (a, c); S1 (g); S2; S3; S4
**EN7864**	*krSi81[Pmyo-3::TIR1::tagBFP::unc-54 3' UTR] I; kr462 III*	*myo-3*	Muscle 2	This work	3; 4 (a, c); S2
**EN7567**	*kr462 III; krSi36[Prab-3::TIR1::tagBFP::unc-54 3' UTR] V*	*rab-3*	Neuron 1	Zhou et al., 2021	3; 4 (a, c); S1 (b); S2; S4
**EN7865**	*kr462 III; krSi140[Prab-3::TIR1::tagBFP::unc-54 3' UTR] IV*	*rab-3*	Neuron 2	This work	3; 4; S2; S3; S4
**EN7691**	*ieSi61[Pges-1::TIR1::mRuby + Cbr-unc-119(+)] II; kr462 III*	*ges-1*	Intestine 1	Zhang et al., 2015	3; 4 (a, c); S1 (h); S2
**EN8173**	*reSi5[Pges-1::TIR1::F2A::mTagBFP2::NLS::AID::tbb-2 3'UTR] I; kr462 III*	*ges-1*	Intestine 2	Guinevere Ashley et al. 2021	3; 4 (a, c); S2
**EN7569**	*krSi63[Pdpy-7::TIR1::tagBFP::unc-54 3' UTR] II; kr462 III*	*dpy-7*	Epidermis 1	Zhou et al., 2021	3; S1 (f); S2
**EN8032**	*kr462 III, krSi53[Pdpy-7::TIR1::tagBFP::unc-54 3' UTR] III*	*dpy-7*	Epidermis 2	This work	3; S2
**EN7861**	*kr462 III; ieSi38[Psun-1::TIR1::mRuby::sun-1 3'UTR + Cbr-unc-119(+)] IV*	*sun-1*	Germline 1	Zhang et al., 2015	3; S1 (e); S2
**EN7862**	*ieSi68[Psun-1::TIR1::mRuby::htp-1 3'UTR + Cbr-unc-119(+)] II; kr462 III*	*sun-1*	Germline 2	Zhang et al., 2015	3; S2
**EN7964**	*ieSi61[Pges-1::TIR1::mRuby + Cbr-unc-119(+)] II; kr462 III; krSi36[Prab-3::TIR1::tagBFP] V*	*ges-1 + rab-3*	Intestine 1 + Neuron 1	Zhang et al., 2015; Zhou et al., 2021	3; S2
**EN7737**	*kr462 III; krSi36[Prab-3::TIR1::tagBFP::unc-54 3' UTR] V, krSi55[Pmyo-3::TIR1::tagBFP::unc-54 3' UTR] V*	*rab-3 + myo-3*	Neuron 1 + Muscle 1	Zhou et al., 2021	4 (a, c)
**EN8492**	*kr462 III; krSi52[Punc-17::TIR1::BFP::unc-54 3' UTR]*	*unc-17*	Cholinergic neuron	This work	4 (b)
**EN8634**	*kr462 III; krSi232[Punc-17::TIR1::BFP::unc-54 3' UTR]*	*unc-17*	Cholinergic neuron	This work	4 (b)
**EN8635**	*kr462 III; krSi233[Punc-17::TIR1::BFP::unc-54 3' UTR]*	*unc-17*	Cholinergic neuron	This work	4 (b)
**EN8636**	*kr462 III; krSi234[Punc-17::TIR1::BFP::unc-54 3' UTR]*	*unc-17*	Cholinergic neuron	This work	4 (b)
**EN8491**	*kr462 III; krSi51[Punc-47::TIR1::BFP::unc-54 3' UTR] IV*	*unc-47*	GABAergic neuron	This work	4 (b)
**EN8637**	*kr462 III; krSi235[Punc-47::TIR1::BFP::unc-54 3' UTR] IV*	*unc-47*	GABAergic neuron	This work	4 (b)
**EN8638**	*kr462 III; krSi235[Punc-47::TIR1::BFP::unc-54 3' UTR] IV*	*unc-47*	GABAergic neuron	This work	4 (b)
**EN8113**	*kr535[daf-16::linker::wScarlet::linker::3xMYC] I; kr462 III; krSi50[Peft-3::TIR1::tagBFP::unc-54 3' UTR] IV*	*eft-3*	Ubiquitous 1	Zhou et al., 2021	5
**EN8169**	*kr535[daf-16::linker::wScarlet::linker::3xMYC] I; kr462 III; krSi140[Prab-3::TIR1::tagBFP::unc-54 3' UTR] IV*	*rab-3*	Neuron 2	This work	5
**EN8170**	*kr535[daf-16::linker::wScarlet::linker::3xMYC] I; kr462 III; krSi55[Pmyo-3::TIR1::tagBFP::unc-54 3' UTR] V*	*myo-3*	Muscle 1	Zhou et al., 2021	5
**EN8536**	*kr535[daf-16::linker::wScarlet::linker::3xMYC] I; ieSi61[Pges-1::TIR1::mRuby + Cbr-unc-119(+)] II; kr462 III*	*ges-1*	Intestine 1	Zhang et al., 2015	5
**EN8621**	*ot853[daf-16::linker::mNeonGreen::3xFlag::AID] I; kr462 III; krSi140[Prab-3::TIR1::tagBFP::unc-54 3' UTR] IV*	*rab-3*	Neuron 2	This work	6 (a)
**EN8620**	*ot853[daf-16::linker::mNeonGreen::3xFlag::AID] I; kr462 III; krSi55[Pmyo-3::TIR1::tagBFP::unc-54 3' UTR] V*	*myo-3*	Muscle 1	Zhou et al., 2021	6 (b)
**EN8625**	*rrf-3(pk1426)II; kr462 III; krSi55[Pmyo-3::TIR1::tagBFP::unc-54 3' UTR] V*	*myo-3*	Muscle 1	Zhou et al., 2021	6 (c)
**EN7878**	*krSi134[Pmyo-3::tomm-20N::wScarlet::unc-54 3' UTR] I; kr462 III; krSi50[Peft-3::TIR1::tagBFP::unc-54 3' UTR] IV*	*eft-3*	Ubiquitous 1	Zhou et al., 2021	S4
**EN8315**	*krSi134[Pmyo-3::tomm-20N::wScarlet::unc-54 3' UTR] I; kr462 III; krSi55[Pmyo-3::TIR1::tagBFP::unc-54 3' UTR] V*	*myo-3*	Muscle 1	Zhou et al., 2021	S4
**EN8316**	*krSi134[Pmyo-3::tomm-20N::wScarlet::unc-54 3' UTR] I; kr462 III; krSi140[Prab-3::TIR1::tagBFP::unc-54 3' UTR] IV*	*rab-3*	Neuron 2	This work	S4
**EN8489**	*krSi134[Pmyo-3::tomm-20N::wScarlet::unc-54 3' UTR] I; kr462 III; krSi36[Prab-3::TIR1::tagBFP::unc-54 3' UTR] V*	*rab-3*	Neuron 1	Zhou et al., 2021	S4
**EN8490**	*krSi134[Pmyo-3::tomm-20N::wScarlet::unc-54 3' UTR] I; kr462 III; krSi36[Prab-3::TIR1::tagBFP::unc-54 3' UTR] V, krSi55[Pmyo-3::TIR1::tagBFP::unc-54 3' UTR] V*	*rab-3 + myo-3*	Neuron 1 + Muscle 1	Zhou et al., 2021	S4

Other strains used in this study

Strains	Genotypes	Source	Used in figure(s)
**N2**	*wt*	CGC	1; 3; S1 (a); S2
**FS428**	*daf-2(e1370) III (CB1370 outcrossed 6x)*	Mergoud et al., 2018	1 (c, d, e); S2; S3
**EN462**	*kr462[daf-2::linker::AID::linker::mNeonGreen] III*	This work	1; 3; 4; S1 (a); S2; S3
**EN50535**	*kr535[daf-16::linker::wScarlet::linker::3xMYC] I*	This work	5
**OH14125**	*ot853[daf-16::linker::mNeonGreen::3xFlag::AID] I*	Bhattacharya et al., 2019	
**EN8618**	*ot853[daf-16::linker::mNeonGreen::3xFlag::AID] I; kr462 III*	This work	6 (a, b)
**NL2099**	*rrf-3(pk1426)II*	Sijen et al., 2001	
**EN8642**	*rrf-3(pk1426)II; kr462 III*	This work	6 (c)
**EN8313**	*krSi134[Pmyo-3::tomm-20N::wScarlet::unc-54 3' UTR] I; kr462 III*	This work	S4

Table S2: Lifespan data and statistics corresponding to lifespan curves shown in Figures 1, 3 and S2

Experiment number	Corresponding figure	Tissue with DAF-2 degradation (1)	Genotype	Condition (2)	Mean Lifespan	Change compared to the control's mean lifespan if significant (%)	Median Lifespan	Change compared to the control's median lifespan if significant (%)	0.95 LCL (3)	0.95 UCL (3)	# deaths/ total	Adjusted p-values using Log-Rank test (4)
	**Figure 1**											
1	1b	NA	N2	NA	20.2	NA	21	NA	21	22	47/61	NA
1	1b	NA	*kr462 III*	NA	20.9	ns	22	ns	21	22	47/58	vs N2: ns
2	1b	NA	N2	NA	20.3	NA	20	NA	20	23	54/80	NA
2	1b	NA	*kr462 III*	NA	19.4	ns	20	ns	17	20	62/80	vs N2: ns
3	1c	NA	N2	EtOH	23.1	NA	23	NA	21	27	41/80	NA
3	1c	NA	*daf-2(e1370) III*	EtOH	48.1	108.23	50	117.39	50	54	65/80	vs N2: ***; vs Ubiquitous 1: ns
3	1c	NA	*kr462 III; krSi50 IV*	EtOH	24.8	ns	27	ns	23	27	50/80	vs N2: ns
3	1c	Ubiquitous 1	*kr462 III; krSi50 IV*	Aux	46.6	101.73	50	117.39	50	50	78/80	vs N2: ***; vs *kr462 III; krSi50 IV* EtOH: ***
5	1c	NA	N2	EtOH	23	NA	24	NA	21	27	55/78	NA
5	1c	NA	*daf-2(e1370) III*	EtOH	33	43.48	42	75	38	45	54/75	vs N2: ***; vs Ubiquitous 2: ns
5	1c	NA	*kr462 III; krSi45 V*	EtOH	23.6	ns	24	ns	24	27	62/80	vs N2: ns
5	1c	Ubiquitous 2	*kr462 III; krSi45 V*	Aux	32.9	43.04	45	87.50	38	49	66/80	vs N2: ***; vs *kr462 III; krSi50 IV* EtOH: ***
	**Figure 3**											
	**Muscle**											
4	3a	NA	N2	EtOH	23.4	NA	24	NA	21	24	54/80	NA
4	3a	Ubiquitous 1	*kr462 III; krSi50 IV*	Aux	42.3	80.77	52	116.67	48	52	68/81	vs N2: ***
4	3a	Muscle 1	*kr462 III; krSi55 V*	Aux	20.7	-11.54	21	-12.50	21	21	53/80	vs N2: **, vs Ubiquitous 1: ***
5	3b	Control	N2	EtOH	23	NA	24	NA	21	27	55/78	NA
5	3b	Ubiquitous 2	*kr462 III; krSi45 V*	Aux	32.9	43.04	45	87.50	38	49	66/80	vs N2: ***
5	3b	Muscle 2	*krSi81 I; kr462 III*	Aux	23.9	ns	24	ns	21	24	62/81	vs N2: ns
	**Germline**											
6	3c	NA	*kr462 III*	EtOH	23.7	NA	25	NA	23	28	63/78	NA
6	3c	Ubiquitous 2	*kr462 III; krSi45 V*	Aux	29.3	23.63	39	56.00	30	49	74/76	vs *kr462* : ***
6	3c	Germline 1	*kr462 III; ieSi38 IV*	Aux	22.6	ns	23	ns	23	23	79/79	vs *kr462* : ns
5	3d	NA	N2	EtOH	23	NA	24	NA	21	27	55/78	NA
5	3d	Ubiquitous 2	*kr462 III; krSi45 V*	Aux	32.9	43.04	45	87.50	38	49	66/80	vs N2: ***
5	3d	Germline 2	*ieSi68 II; kr462 III*	Aux	23.2	ns	24	ns	21	24	65/79	vs N2: ns
	**Epidermis**											
4	3e	NA	N2	EtOH	23.4	NA	24	NA	21	24	54/80	NA
4	3e	Ubiquitous 1	*kr462 III; krSi50 IV*	Aux	42.3	80.77	52	116.67	48	52	68/81	vs N2: ***
4	3e	Epidermis 1	*krSi63 II; kr462 III*	Aux	20.8	-11.11	21	-12.50	21	21	58/80	vs N2: ***; vs Ubiquitous 1: ***
8	3f	Control	*kr462 III*	Aux	23.3	NA	23	NA	23	25	51/78	NA
8	3f	Ubiquitous 1	*kr462 III; krSi50 IV*	Aux	42.4	81.97	49	113.04	46	49	68/80	vs *kr462* : ***
8	3f	Epidermis 2	*kr462, krSi53 III*	Aux	25.8	10.73	25	8.70	25	28	49/78	vs *kr462* : ***; vs Ubiquitous 1: ***
	**Intestine**											
4	3g	NA	N2	EtOH	23.4	NA	24	NA	21	24	54/80	NA
4	3g	Ubiquitous 1	*kr462 III; krSi50 IV*	Aux	42.3	80.77	52	116.67	48	52	68/81	vs N2: ***
4	3g	Intestine 1	*ieSi61 II; kr462 III*	Aux	40.7	73.93	41	70.83	41	41	33/63	vs N2: ***; vs Ubiquitous 1: ***
8	3h	Control	*kr462 III*	Aux	23.3	NA	23	NA	23	25	51/78	NA
8	3h	Ubiquitous 1	*kr462 III; krSi50 IV*	Aux	42.4	81.97	49	113.04	46	49	68/80	vs *kr462* : ***
8	3h	Intestine 2	*reSi5 I; kr462 III*	Aux	37.2	59.66	42	82.61	37	44	43/79	vs *kr462* : ***; vs Ubiquitous 1: ***
	**Neurons**											
4	3i	NA	N2	EtOH	23.4	NA	24	NA	21	24	54/80	NA
4	3i	Ubiquitous 1	*kr462 III; krSi50 IV*	Aux	42.3	80.77	52	116.67	48	52	68/81	vs N2: ***
4	3i	Neuron 1	*kr462 III; krSi36 V*	Aux	32.3	38.03	34	41.67	31	38	65/80	vs N2: ***; vs Ubiquitous 1: ***
5	3j	NA	N2	EtOH	23	NA	24	NA	21	27	55/78	NA
5	3j	Ubiquitous 2	*kr462 III; krSi45 V*	Aux	32.9	43.04	45	87.50	38	49	66/80	vs N2: ***
5	3j	Neuron 2	*kr462 III; krSi140 IV*	Aux	32	39.13	42	75.00	35	42	76/84	vs N2: ***; vs Ubiquitous 2: ***
	**Neurons + Intestine**											
7	3k	NA	*kr462 III*	EtOH	22.2	NA	25	NA	21	25	55/73	NA
7	3k	Ubiquitous 1	*kr462 III; krSi50 IV*	Aux	39	75.68	42	68.00	42	46	75/87	vs *kr462* : ***
7	3k	Intestine 1	*ieSi61 II; kr462 III*	Aux	35.4	59.46	35	40.00	35	39	62/68	vs *kr462* : ***; vs Ubiquitous 1: ***
7	3k	Intestine 1 + Neuron 1	*ieSi61 II; kr462 III; krSi36 V*	Aux	33.2	49.55	35	40.00	32	35	77/81	vs *kr462:* ***; vs Ubiquitous 1: ***; vs Intestine 1: ns

Experiment number	Corresponding figure	Tissue with DAF-2 degradation (1)	Genotype	Condition (2)	Mean Lifespan	Change compared to the control's mean lifespan if significant (%)	Median Lifespan	Change compared to the control's median lifespan if significant (%)	0.95 LCL (3)	0.95 UCL (3)	# deaths/ total	Adjusted p-values using Log-Rank test (4)
	**Figure S2**											
3	2a	NA	N2	EtOH	23.1	NA	23	NA	21	27	41/80	NA
3	2a	NA	N2	Aux	22.5	ns	23	ns	21	27	46/80	vs N2 EtOH: ns
10	2b	NA	N2	EtOH	24.9	NA	27	NA	22	29	66/79	NA
10	2b	NA	N2	Aux	25.1	ns	24	ns	24	27	66/80	vs N2 EtOH: ns
10	2b	NA	*kr462 III*	EtOH	25.3	ns	27	ns	24	27	68/81	vs N2 EtOH: ns
10	2b	NA	*kr462 III*	Aux	23	-9.09 (vs *kr462* EtOH)	24	-11.11 (vs *kr462* EtOH)	20	27	60/80	vs N2 Aux: ns; vs *kr462* EtOH: *
9	2c	NA	N2	EtOH	23.1	NA	24	NA	22	27	56/80	NA
9	2c	NA	N2	Aux	20.5	-11.25 (vs N2 EtOH)	20	-16.67 (vs N2 EtOH)	20	22	61/80	vs N2 EtOH: *
9	2c	NA	*kr462 III*	EtOH	23.5	ns	24	ns	22	24	55/80	vs N2 EtOH: ns
9	2c	NA	*kr462 III*	Aux	21.1	-10.21 (vs *kr462* EtOH)	22	-8.33 (vs *kr462* EtOH)	20	24	55/80	vs N2 Aux: ns; vs *kr462* EtOH: *
9	2c	NA	*daf-2(e1370) III*	EtOH	40.5	91.94	48	118.18	45	48	63/80	vs N2 EtOH: ***; vs Ubiquitous 1: **; vs Ubiquitous 2: ***
9	2c	Ubiquitous 1	*kr462 III; krSi50 IV*	Aux	40.3	91.00	48	118.18	48	52	74/80	vs *kr462* Aux: ***
9	2c	Ubiquitous 2	*kr462 III; krSi45 V*	Aux	40.2	90.52	52	136.36	48	55	55/60	vs *kr462* Aux: ***; vs Ubiquitous 1: ns
	**Muscle**											
10	2d	NA	*kr462 III*	Aux	23	NA	24	NA	20	27	60/80	NA
10	2d	Muscle 1	*kr462 III; krSi55 V*	Aux	23.5	ns	24	ns	22	27	60/80	vs *kr462* : ns
10	2d	Muscle 2	*krSi81 I; kr462 III*	Aux	24.3	ns	24	ns	22	27	49/79	vs *kr462* : ns
	**Germline**											
10	2e	NA	*kr462 III*	Aux	23	NA	24	NA	20	27	60/80	NA
10	2e	Germline 1	*kr462 III; ieSi38 IV*	Aux	26.3	14.35	27	12.50	27	29	63/80	vs *kr462* : *
10	2e	Germline 2	*ieSi68 II; kr462 III*	Aux	24.7	ns	24	ns	24	27	64/80	vs *kr462* : ns
	**Epidermis**											
10	2f	NA	*kr462 III*	Aux	23	NA	24	NA	20	27	60/80	NA
10	2f	Epidermis 1	*krSi63 II; kr462 III*	Aux	22.7	ns	22	ns	20	27	30/39	vs *kr462* : ns
10	2f	Epidermis 2	*kr462, krSi53 III*	Aux	25.8	12.17	27	12.50	24	29	67/80	vs *kr462* : *
11	2g	NA	*kr462 III*	Aux	22.7	NA	22	NA	22	24	55/80	NA
11	2g	Epidermis 1	*krSi63 II; kr462 III*	Aux	22.9	ns	22	ns	22	24	63/80	vs *kr462* : ns
	**Intestine**											
6	2h	NA	*kr462 III*	EtOH	23.7	NA	25	NA	23	28	63/78	NA
6	2h	Ubiquitous 2	*kr462 III; krSi45 V*	Aux	29.3	23.63	39	56.00	30	49	74/76	vs *kr462* : ***
6	2h	Intestine 1	*ieSi61 II; kr462 III*	Aux	31.3	32.07	35	40.00	35	37	67/82	vs *kr462* : ***; vs Ubiquitous 2: ***
	**Neurons**											
7	2i	NA	*kr462 III*	EtOH	22.2	NA	25	NA	21	25	55/73	NA
7	2i	Ubiquitous 1	*kr462 III; krSi50 IV*	Aux	39	75.68	42	68.00	42	46	75/87	vs *kr462* : ***
7	2i	Ubiquitous 2	*kr462 III; krSi45 V*	Aux	45	102.70	53	112.00	53	60	60/76	vs *kr462* : ***; vs Ubiquitous 1: ***
7	2i	Neuron 1	*kr462 III; krSi36 V*	Aux	27.2	22.52	25	0.00	25	32	51/56	vs *kr462* : ***; vs Ubiquitous 1: ***
7	2i	Neuron 2	*kr462 III; krSi140 IV*	Aux	31.4	41.44	32	28.00	32	35	59/73	vs *kr462* : ***; vs Ubiquitous 1: ***
	**Neurons + Intestine**											
8	2j	NA	*kr462 III*	Aux	23.3	NA	23	NA	23	25	51/78	NA
8	2j	Ubiquitous 1	*kr462 III; krSi50 IV*	Aux	42.4	81.97	49	113.04	46	49	68/80	vs *kr462* : ***
8	2j	Intestine 1	*ieSi61 II; kr462 III*	Aux	33.4	43.35	35	52.17	32	37	52/73	vs *kr462* : ***; vs Ubiquitous 1: ***
8	2j	Neuron 1	*kr462 III; krSi36 V*	Aux	33.6	44.21	37	60.87	35	37	70/80	vs *kr462* : ***; vs Ubiquitous 1: ***
8	2j	Intestine 1 + Neuron 1	*ieSi61 II; kr462 III; krSi36 V*	Aux	35.1	50.64	37	60.87	32	39	69/80	vs *kr462* : ***; vs Ubiquitous 1: ***; vs Neuron 1: ns

1) Depending on the experiments, different controls were used: N2 and *daf-2(kr462)* strains have a similar lifespan (in the presence or absence of 1 mM auxin).
In half of the control tests, the presence of auxin resulted in a significant 10 % decrease in the lifespan of both strains (experiments number 1, 2, 3, 9 and 10).
The use of worms carrying the *kr462* and *TIR1* alleles in the absence of auxin as controls was avoided, as low constitutive activity of TIR1 has been reported (data not shown and Hills-Muckey et al., 2022)

(2) EtOH: ethanol; Aux: 1 mM auxine

(3) LCL: Lower Confidence Limit; UCL: Upper Confidence Limit

(4) Comparisons between strains in the same experiment (see first column, "experiment number")
Adjusted p-value: ns: not significant; * < 0.05; ** < 0.01; *** < 0.001

Summary of lifespan replicates per strain and results

Tissue	Allele	Number of replicates	Number of times with significant difference compared to the control	Change compared to the control's mean lifespan if significant (%)	Change compared to the control's median lifespan if significant (%)	Experiment number
Ubiquitous	Ubiquitous 1 (*krSi50*)	5	5	101.73	117.39	3
				80.77	116.67	4
				75.68	68.00	7
				81.97	113.04	8
				91.00	118.18	9
	Ubiquitous 2 (*krSi45*)	4	4	43.04	87.50	5
				23.63	56.00	6
				102.70	112.00	7
				90.52	136.36	9
Intestine	Intestine 1 (*ieSi61*)	4	4	73.93	70.83	4
				32.07	40.00	6
				59.46	40.00	7
				43.35	52.17	8
	Intestine 2 (*reSi5*)	1	1	59.66	82.61	8
Neuron	Neuron 1 (*krSi36*)	3	3	38.03	41.67	4
				22.52	0.00	7
				44.21	60.87	8
	Neuron 2 (*krSi140*)	2	2	39.13	75.00	5
				41.44	28.00	7
Intestine + Neuron	Intestine 1 (*ieSi61*) + Neuron 1 (*krSi36*)	2	2	49.55	40.00	7
				50.64	60.87	8
Muscle	Muscle 1 (*krSi55*)	2	1	-11.54	-12.50	4
				ns	ns	10
	Muscle 2 (*krSi81*)	2	0	ns	ns	5
				ns	ns	10
Germline	Germline 1 (*ieSi38*)	2	1	ns	ns	6
				14.35	12.50	10
	Germline 2 (*ieSi68*)	2	0	ns	ns	5
				ns	ns	10
Epidermis	Epidermis 1 (*krSi63*)	3	1	-11.11	-12.50	4
				ns	ns	10
				ns	ns	11
	Epidermis 2 (*krSi53*)	2	2	10.73	8.70	8
				12.17	12.50	10

Table S2: Lifespan data and statistics corresponding to lifespan curves in presence of paraquat and auxine shown in Figures 3 and S2

Experiment number	Corresponding figure	Tissue with DAF-2 degradation	Genotype	Mean Lifespan	Change compared to the control's mean lifespan if significant (%)	Median Lifespan	Change compared to the control's median lifespan if significant (%)	0.95 LCL (1)	0.95 UCL (1)	# deaths/ total	Adjusted p-values using Log-Rank test (2)
	**Figure 3**										
	**Intestine**										
12	3l	NA	*kr462 III*	2.6	NA	2.6	NA	2.6	2.6	53/75	NA
12	3l	Ubiquitous 1	*kr462 III; krSi50 IV*	3.9	50.00	4.7	80.77	4.7	6	69/75	vs *kr462* : ***
12	3l	Ubiquitous 2	*kr462 III; krSi45 V*	3.9	50.00	6	130.77	6	6	73/75	vs *kr462* : ***; vs Ubiquitous 1: ***
12	3l	Intestine 1	*ieSi61 II; kr462 III*	3.5	34.62	3.6	38.46	3.6	3.6	67/75	vs *kr462* : ***; vs Ubiquitous 1: ***
12	3l	Intestine 2	*reSi5 I; kr462 III*	3.2	23.08	3	15.38	3	3.6	71/75	vs *kr462* : ***; vs Ubiquitous 1: ***
	**Neurons**										
13	3m	NA	*kr462 III*	2.6	NA	2.6	NA	2.6	2.6	84/100	NA
13	3m	Ubiquitous 1	*kr462 III; krSi50 IV*	3.9	50.00	4.2	61.54	4.2	4.8	81/100	vs *kr462* : ***
13	3m	Neuron 1	*kr462 III; krSi36 V*	2.2	-15.38	2	-23.08	2	2	95/100	vs *kr462* : ***; vs Ubiquitous 1: ***
13	3m	Neuron 2	*kr462 III; krSi140 IV*	2.9	11.54	2.6	0.00	2.6	3	89/97	vs *kr462* : ***; vs Ubiquitous 1: ***
	**Intestine + Neurons**										
14	3n	NA	*kr462 III*	2.8	NA	3	NA	2.5	3	74/91	NA
14	3n	Ubiquitous 1	*kr462 III; krSi50 IV*	4.4	57.14	5	66.67	5	5.5	73/90	vs *kr462* : ***
14	3n	Intestine 1	*ieSi61 II; kr462 III*	3.7	32.14	3.5	16.67	3.5	3.5	72/92	vs *kr462* : ***; vs Ubiquitous 1: ***
14	3n	Neuron 1	*kr462 III; krSi36 V*	2.3	-17.86	2.5	-16.67	2.5	2.5	75/90	vs *kr462* : ***; vs Ubiquitous 1: ***
14	3n	Intestine 1 + Neuron 1	*ieSi61 II; kr462 III; krSi36 V*	3.1	10.71	3	0.00	3	3.5	71/91	vs *kr462* : ***; vs Ubiquitous 1: ***; vs Intestine 1: ***
	**Figure S2**										
	**Intestine + Neurons**										
13	2k	NA	*kr462 III*	2.6	NA	2.6	NA	2.6	2.6	84/100	NA
13	2k	Ubiquitous 1	*kr462 III; krSi50 IV*	3.9	50.00	4.2	61.54	4.2	4.8	81/100	vs *kr462* : ***
13	2k	Intestine 1	*ieSi61 II; kr462 III*	3.5	34.62	3.6	38.46	3.6	3.6	96/100	vs *kr462* : ***; vs Ubiquitous 1: ***
15	2l	NA	*kr462 III*	2.7	NA	2.7	NA	2.7	2.7	88/100	NA
15	2l	Ubiquitous 1	*kr462 III; krSi50 IV*	4.2	55.56	4.2	55.56	4.2	4.2	97/100	vs *kr462* : ***
15	2l	Intestine 2	*reSi5 I; kr462 III*	3.5	29.63	3.8	40.74	3.8	3.8	95/100	vs *kr462* : ***; vs Ubiquitous 1: ***
15	2l	Neuron 2	*kr462 III; krSi140 IV*	3.4	25.93	3.2	18.52	3.2	3.2	87/100	vs *kr462* ***; vs Ubiquitous 1: ***
14	2m	NA	*kr462 III*	2.8	NA	3	NA	2.5	3	74/91	NA
14	2m	Ubiquitous 1	*kr462 III; krSi50 IV*	4.4	57.14	5	66.67	5	5.5	73/90	vs *kr462* : ***
14	2m	Neuron 2	*kr462 III; krSi140 IV*	3.2	14.29	3	0.00	2.5	3	49/92	vs *kr462* : **; vs Ubiquitous 1: ***
15	2n	NA	*kr462 III*	2.7	NA	2.7	NA	2.7	2.7	88/100	NA
15	2n	Ubiquitous 1	*kr462 III; krSi50 IV*	4.2	55.56	4.2	55.56	4.2	4.2	97/100	vs *kr462* : ***
15	2n	Intestine 1	*ieSi61 II; kr462 III*	3.6	33.33	3.8	40.74	3.8	3.8	98/100	vs *kr462* : ***; vs Ubiquitous 1: ***
15	2n	Neuron 1	*kr462 III; krSi36 V*	2.5	-7.41	2.7	0.00	2.7	2.7	98/100	vs *kr462* : ***; vs Ubiquitous 1: ***
15	2n	Intestine 1 + Neuron 1	*ieSi61 II; kr462 III; krSi36 V*	3.2	18.52	3.2	18.52	3.2	3.2	98/100	vs *kr462* : ***; vs Ubiquitous 1: ***; vs Intestine 1: ***
	**Muscle**										
13	2o	NA	*kr462 III*	2.6	NA	2.6	NA	2.6	2.6	84/100	NA
13	2o	Ubiquitous 1	*kr462 III; krSi50 IV*	3.9	50.00	4.2	61.54	4.2	4.8	81/100	vs *kr462* : ***
13	2o	Ubiquitous 2	*kr462 III; krSi45 V*	4.2	61.54	5.6	115.38	5.6	6.8	78/100	vs *kr462* : ***; vs Ubiquitous 1: ***
13	2o	Muscle 1	*kr462 III; krSi55 V*	2.6	ns	2.6	ns	2.6	2.6	92/99	vs *kr462* : ns
13	2o	Muscle 2	*krSi81 I; kr462 III*	2.6	ns	2.6	ns	2.6	2.6	97/100	vs *kr462* : ns
14	2p	NA	*kr462 III*	2.8	NA	3	NA	2.5	3	74/91	NA
14	2p	Ubiquitous 1	*kr462 III; krSi50 IV*	4.4	57.14	5	66.67	5	5.5	73/90	vs *kr462* : ***
14	2p	Muscle 1	*kr462 III; krSi55 V*	2.7	-3.70	2.5	-16.67	2.5	3	63/92	vs *kr462* : *; vs Ubiquitous 1: ***

(1) LCL: Lower Confidence Limit; UCL: Upper Confidence Limit

(2) Comparisons between strains in the same experiment (see first column, "experiment number")
Adjusted p-value: ns: not significant; * < 0.05; ** < 0.01; *** < 0.001

Summary of lifespan replicates in presence of paraquat per strain and results

Tissue	Allele	Number of replicates	Number of times with significant difference compared to the control	Change compared to the control's mean lifespan if significant (%)	Change compared to the control's median lifespan if significant (%)	Experiment number
Ubiquitous	Ubiquitous 1 (*krSi50*)	4	4	50.00	80.77	12
				50.00	61.54	13
				57.14	66.67	14
				55.56	55.56	15
	Ubiquitous 2 (*krSi45*)	2	2	50.00	130.77	12
				61.54	115.38	13
Intestine	Intestine 1 (*ieSi61*)	4	4	34.62	38.46	12
				34.62	38.46	13
				32.14	16.67	14
				33.33	40.74	15
	Intestine 2 (*reSi5*)	2	2	23.08	15.38	12
				29.63	40.74	15
Neuron	Neuron 1 (*krSi36*)	3	3	-15.38	-23.08	13
				-17.86	-16.67	14
				-7.41	0.00	15
	Neuron 2 (*krSi140*)	3	3	11.54	0.00	13
				14.29	0.00	14
				25.93	18.52	15
Intestine + Neuron	Intestine 1 (*ieSi61*) + Neuron 1 (*krSi36*)	2	2	10.71	0.00	14
				18.52	18.52	15
Muscle	Muscle 1 (*krSi55*)	2	1	ns	ns	13
				-3.70	-16.67	14
	Muscle 2 (*krSi81*)	1	0	ns	ns	13

Table S3: dauer percentage

TIR1 strain (see Table S1)	Percentage of dauer (n; N)	
	+ EtOH	+ Auxin
Ubiquitous 1	0 (1640; 5)	100 (605; 5)
Ubiquitous 2	0 (112; 3)	100 (68; 3)
Neuron 1	0 (761; 3)	0 (546; 3)
Neuron 2	0 (164; 3)	0 (157; 3)
Muscle 1	0 (626; 3)	0 (633; 3)
Intestine 1	0 (1153; 4)	0 (984; 4)
Epidermis 1	0 (670; 2)	0 (421; 2)
Germline 2	0 (189; 3)	0 (143; 3)
Neuron 1 + Muscle 1	0 (427; 2)	0 (366; 2)
Neuron 1 + Intestine 1	0 (201; 3)	0 (170; 3)

Table S4: DAF-16 nuclear translocation

		Percentage of worms with strong DAF-16::wrmSCARLET nuclear signal (n)		
		Intestine	Neurons	Muscles
DAF-2 degradation	Control	0 (22)	0 (25)	0 (22)
	Ubiquitous	100 (31)	100 (28)	100 (32)
	Intestine	100 (21)	0 (21)	100 (22)
	Neuron	0 (25)	100 (28)	0 (25)
	Muscle	0 (25)	0 (25)	100 (25)

Table S5

List of generated plasmids

Plasmid	Description	Usage
pCR12	*last 280 bp of daf-2::flexible linker::AID::flexible linker::mNeonGreen::daf-2 3' UTR first 347 bp*	Repair template to create EN462 CRISPR knock-in strain
pCV06	*Punc-17::TIR1-TagBFP::unc-54 3'UTR*	Minimal Mos 1 vector for *Punc-17::TIR1-TagBFP* insertion; used to create *krSi52* allele; contains a neomycin-resistant cassette
pCV07	*Punc-47::TIR1-TagBFP::unc-54 3'UTR*	Minimal Mos 1 vector for *Punc-47::TIR1-TagBFP* insertion; used to create *krSi51* allele; contains a neomycin-resistant cassette
pBB16	*Pmyo-3::tomm-20N::wScarlet::unc-54 3' UTR*	Minimal Mos 1 vector for *Pmyo-3::tomm-20N::wSCARLET* insertion; used to create *krSi134* allele; contains a neomycin-resistant cassette

List of generated miniMos single-copy insertion alleles

Allele name	Construct	Plasmid used
krSi45	*Peft-3::TIR1::tagBFP::unc-54 3'UTR V*	pCV09 (Zhou et al., 2021)
krSi81	*Pmyo-3::TIR1::tagBFP::unc-54 3'UTR I*	pCV04 (Zhou et al., 2021)
krSi140	*Prab-3::TIR1::tagBFP::unc-54 3'UTR IV*	pCV05 (Zhou et al., 2021)
krSi53	*Pdpy-7::TIR1::tagBFP::unc-54 3'UTR III*	pCV08 (Zhou et al., 2021)
krSi52	*Punc-17::TIR1::BFP::unc-54 3'UTR*	pCV06 (this work)
krSi232	*Punc-17::TIR1::BFP::unc-54 3'UTR*	pCV06 (this work)
krSi233	*Punc-17::TIR1::BFP::unc-54 3'UTR*	pCV06 (this work)
krSi234	*Punc-17::TIR1::BFP::unc-54 3'UTR*	pCV06 (this work)
krSi51	*Punc-47::TIR1::BFP::unc-54 3'UTR IV*	pCV07 (this work)
krSi235	*Punc-47::TIR1::BFP::unc-54 3'UTR*	pCV07 (this work)
krSi236	*Punc-47::TIR1::BFP::unc-54 3'UTR*	pCV07 (this work)
krSi134	*Pmyo-3::tomm-20N::wScarlet::unc-54 3' UTR I*	pBB16 (this work)
